# **γ**-Secretase-Dependent Proteolysis of Transmembrane Domain of Amyloid Precursor Protein: Successive Tri- and Tetrapeptide Release in Amyloid **β**-Protein Production

**DOI:** 10.1155/2012/591392

**Published:** 2012-12-31

**Authors:** Mako Takami, Satoru Funamoto

**Affiliations:** ^1^Department of Neuropathology, Graduate School of Life and Medical Sciences, Doshisha University, Kyotanabe, Kyoto 610-0934, Japan; ^2^Pharma Eight Co. Ltd., Kyoto, Kyoto 602-0841, Japan

## Abstract

**γ**-Secretase cleaves the carboxyl-terminal fragment (**β**CTF) of APP not only in the middle of the transmembrane domain (**γ**-cleavage), but also at sites close to the membrane/cytoplasm boundary (**ε**-cleavage), to produce the amyloid **β** protein (A**β**) and the APP intracellular domain (AICD), respectively. The AICD49–99 and AICD50–99 species were identified as counterparts of the long A**β** species A**β**48 and A**β**49, respectively. We found that A**β**40 and AICD50–99 were the predominant species in cells expressing wild-type APP and presenilin, whereas the production of A**β**42 and AICD49–99 was enhanced in cells expressing familial Alzheimer's disease mutants of APP and presenilin. These long A**β** species were identified in cell lysates and mouse brain extracts, which suggests that **ε**-cleavage is the first cleavage of **β**CTF to produce A**β** by **γ**-secretase. Here, we review the progress of research on the mechanism underlying the proteolysis of the APP transmembrane domain based on tri- and tetrapeptide release.

## 1. Introduction

The amyloid precursor protein (APP) is a type I membrane protein. After ectodomain shedding by *β*-secretase, the carboxyl-terminal fragment (*β*CTF) of APP becomes a direct substrate of *γ*-secretase and is processed into the amyloid *β* protein (A*β*) and the APP intracellular domain (AICD) [1–5]. *γ*-secretase is an enigmatic protease composed of presenilin 1/2, nicastrin, Aph-1, and Pen-2 that catalyzes proteolysis in the hydrophobic environment of the lipid bilayer [6–15]. Currently, over 50 molecules are reported as *γ*-secretase substrates, which reflects the physiological importance of this enzyme [16]. For instance, the Notch receptor on the plasma membrane is cleaved by *γ*-secretase upon ligand binding and the liberated Notch intracellular domain (NICD) translocates into the nucleus and activates the expression of transcription factors to suppress neuronal differentiation [17, 18]. This indicates that inhibition of *γ*-secretase for suppression of A*β* production causes harmful side effects. To avoid this risk in anti-Alzheimer's disease (AD) therapeutics, it is very important to elucidate the molecular mechanism underlying *γ*-secretase-dependent proteolysis. Recently, it was revealed that *γ*-secretase forms a hydrophilic pore and three water-accessible cavities [19–23]. Here, we review the progress of research on the mechanism underlying the proteolysis of the transmembrane domain of *β*CTF.

## 2. Discovery of *ε*-Cleavage during APP Processing

**Figure 1:**
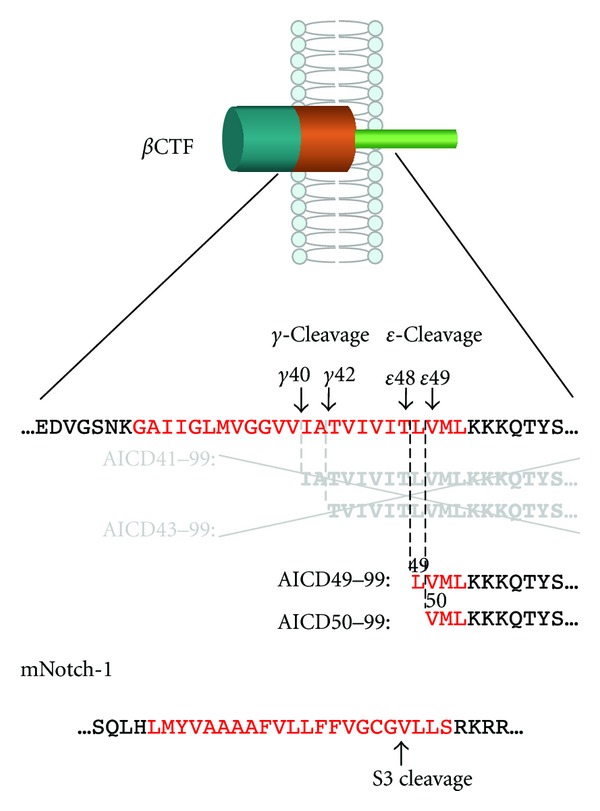
*β*CTF is cleaved at the membrane-cytoplasm boundary and not in the middle of the transmembrane domain (*ε*-cleavage), to release the AICD49–99 and AICD50–99 species. The production of AICD species was inhibited in the presence of a *γ*-secretase inhibitor. *ε*-Cleavage is analogous to the S3 cleavage of mNotch-1. Red indicates the transmembrane domain.

After the *β*-secretase-dependent cleavage of APP, the ectodomain of APP is released into the extracellular space and *β*CTF (as a stub in the lipid bilayer) is the direct substrate of *γ*-secretase [2, 3, 24]. *β*CTF is composed of 99 amino acids and is eventually processed into the 38–43-residue-long A*β*, suggesting that the counterparts of those A*β* species should contain 56–61 residues [4, 25–29]. However, 50-51-residue-long AICDs were identified that correspond to residues 49–99 and 50–99 of *β*CTF (AICD49–99 and AICD50–99), instead of 56–61-residue-long species (Figure 1) [30–32]. These AICD species were suppressed by L-685,458, a transition state analogue *γ*-secretase inhibitor, and by expression of a dominant-negative mutant of presenilin (PS), suggesting that *γ*-secretase cleaves *β*CTF not only in the middle of the transmembrane domain (*γ*-cleavage), but also at sites close to the membrane/cytoplasm boundary (*ε*-cleavage), releasing AICD49–99 and AICD50–99. *ε*-Cleavage sites are analogues of the Notch S3 cleavage site, which is located at the membrane, near the cytoplasm (Figure 1). Cleavages similar to the APP *ε*-cleavage were identified in other proteins, such as amyloid precursor-like protein 1 (APLP-1), APLP-2, CD44, Delta 1, E-cadherin, ErbB4, and LRP1 [30, 33–37]. It is reasonable to consider that the water molecules required for proteolysis have access to the catalytic center of *γ*-secretase from the cytoplasm, rather than from the extracellular space, and that *ε*-cleavage precedes *γ*-cleavage during APP processing.

## 3. Relationship between *γ*- and *ε*-Cleavage

**Figure 2:**
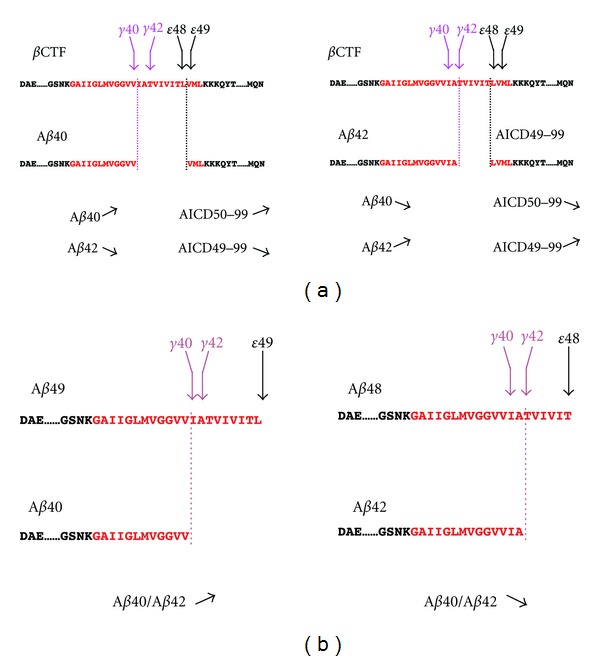
Relationship between *γ*- and *ε*-cleavage. (a) Cells expressing wild-type PS or APP predominantly produce A*β*40 and AICD50–99, while cells expressing a FAD mutant of PS or APP exhibited increased proportion of A*β*42 and AICD49–99. (b) Expression of A*β*49 results in an increase in A*β*40/A*β*42 ratio, whereas expression of A*β*48 leads to opposite results. ↗ increase, ↘ decrease.

CHO cells expressing familial AD (FAD) mutants of PS or APP increase production ratio of A*β*42 (A*β*43) to A*β*40 compared to cells expressing wild-type PS or APP these longer A*β* species are more hydrophobic and more prone to form neurotoxic aggregates. CHO cells expressing wild-type PS preferentially release AICD50–99, whereas those expressing a subset of familial AD (FAD) mutants of PS or APP exhibit an increased proportion of AICD49–99 (Figure 2(a)) [42]. As those FAD mutations cause an increase in the A*β*42/A*β*40 ratio, a potential link between *γ*- and *ε*-cleavage was assumed. To test this, we expressed A*β*49 and A*β*48, which are potential counterparts of AICD50–99 and AICD49–99, respectively, in CHO cells. The cells expressing A*β*49 predominantly secreted A*β*40, whereas those expressing A*β*48 exhibited a significantly increased proportion of A*β*42/A*β*40 (Figure 2(b)) [43]. These data indicate that *ε*-cleavage sites determine the preference for *γ*- and *ε*-cleavage sites to produce A*β*40 and A*β*42. Long A*β* species, A*β*49 and A*β*48, have been identified in cell lysates and mouse brain extracts, which suggests that *ε*-cleavage is the first cleavage of *β*CTF to produce A*β* by *γ*-secretase [44]. On the other hand, *ε*-cleavage can be considered as endopeptidase activity of *γ*-secretase. FAD mutations did not consistently impair the endopeptidase activity on APP, Notch, ErbB4, and N-Cadherin, but altered *γ*-cleavage of APP, especially fourth cleavage to produce A*β*40 and A*β*38 from A*β*43 and A*β*42, respectively [45]. Such dissociation between *ε*-cleavage and *γ*-cleavage was also proposed by Quintero-Monzon et al. [46].

## 4. Tripeptide Hypothesis

Treatment with N-[N-(3,5-difluorophenacetyl)-L-alanyl]-(S)-phenylglycine t-butyl ester (DAPT), a *γ*-secretase inhibitor, suppressed extracellular A*β* in cells expressing APP [47]. The levels of the intracellular A*β*40 and A*β*42 species also decreased after DAPT treatment; however, intracellular A*β*43 and A*β*46 increased in a dose-dependent manner [44, 48, 49]. Tryptophan substitutions of *γ*-cleavage site (41–43) of APP attenuated A*β* secretion, but accumulated A*β*45 species in cell lysate. Tryptophan substitutions of *ε*-cleavage site (48–52) of APP decreased A*β* production and allowed longer AICD46–99 production. Tryptophan substitutions of *ξ*-cleavage site (45–47) also suppressed A*β* production. These substitution studies also implied successive cleavage of APP for A*β* production after *ε*-cleavage [50]. 

**Figure 3:**
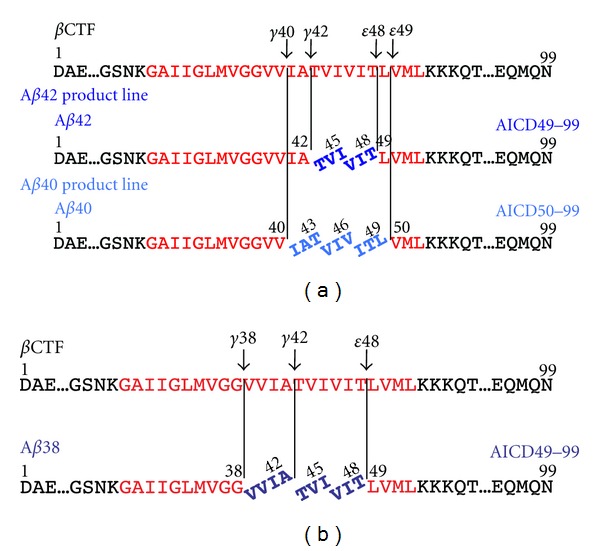
Tri- and tetrapeptide release from *β*CTF. (a) Upon *ε*-cleavage at *ε*48, *γ*-secretase releases the VIT and TVI tripeptides successively to produce A*β*42. (b) In the A*β*40 product line, after *ε*-cleavage at *ε*49, *β*CTF is converted into A*β*40 by releasing ITL, VIV, and IAT. A*β*42 is a direct substrate during A*β*38 production, which acts by releasing the VVIA tetrapeptide.

*γ*-Secretase containing mature nicastrin accumulates in lipid rafts, which indicates that active *γ*-secretase mainly localizes to the lipid raft of cells [51]. Lipid rafts are an ideal material to investigate A*β* production in the membrane environment. A*β*46 was the dominant species in a lipid raft isolated from DAPT-treated cells. Interestingly, incubating this lipid raft in the absence of DAPT resulted in production of A*β*40 and A*β*43, but not of A*β*42 [52]. These data suggest that A*β*46 is mainly converted into A*β*40 by releasing VIV and IAT tripeptides (successive tripeptide release, tripeptide hypothesis; A*β*40 product line) (Figure 3(a)). On the other hand, CHO cells expressing an FAD mutant of presenilin 2 exhibited a decrease in intracellular A*β*42 and a concomitant increase in intracellular A*β*45 levels in the presence of DAPT, suggesting that A*β*45 is a precursor of A*β*42 by releasing TVI (A*β*42 product line) (Figure 3(a)) [53]. It is reasonable to consider that two major product lines lead to A*β*40 and A*β*42 production (Figure 3(a)).

## 5. Identification of Tri- and Tetrapeptides Released from *β*CTF

The most effective approach to confirm tripeptide release from *β*CTF is the identification of those tripeptides directly in the reaction mixture of A*β* production. CHAPSO soluble *γ*-secretase was isolated and incubated with the *β*CTF substrate. LC-MS/MS analysis identified five major tripeptides, and *γ*-secretase inhibitors abolished the production of these molecules. ITL, VIV, and IAT were predicted tripeptides in the A*β*40 product line (Figure 3(a)). The amounts of A*β*40 and A*β*43 in the reaction mixture, as assessed using Western blotting, corresponded roughly to the predicted A*β*40 and A*β*43 levels, respectively [38]. VIT and TVI were also detected in the A*β*42 product line, as predicted (Figure 3(a)). Interestingly, the VVIA tetrapeptide was detected in the reaction mixture only in the absence of *γ*-secretase inhibitors (Figure 3(b)). We postulated that VVIA was released from A*β*42 to produce A*β*38. No significant difference was detected between the level of A*β*42 by Western blot quantification and that by LC-MS/MS quantitative estimation. These results indicate that *γ*-secretase releases tri- and tetrapeptides successively upon *ε*-cleavage of *β*CTF, to produce A*β* species. These tri- and tetrapeptides released from *β*CTF were detected even in the lipid raft fraction (Takami, unpublished observation).

## 6. Is Tripeptide Release a General Property of Substrate Cleavage by *γ*-Secretase?

Successive tripeptide release was observed in *β*CTF processing by *γ*-secretase. We also found that *γ*-secretase released tri- and tetrapeptides successively from *α*CTF substrate (Takami, unpublished observation). Recently, tripeptide spacing of endoproteolysis on presenilin has been reported [54]. These suggest that successive tri- and tetrapeptide release is a general property of *γ*-secretase-mediated intramembrane proteolysis.

**Figure 4:**
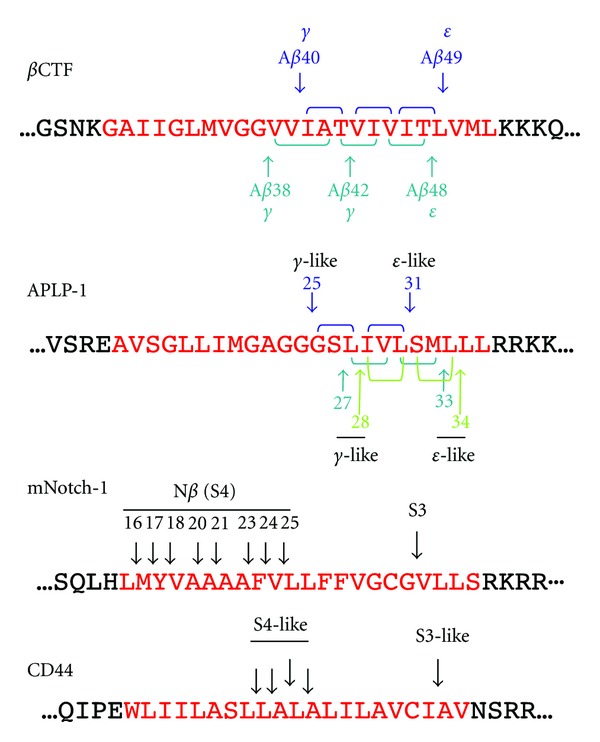
Multiple cleavage sites on the transmembrane domain of *γ*-secretase substrates. APP [38], APLP-1 [30, 39], mNotch-1 [40], and CD44 [41].

Yanagida et al. reported that APLP-1 was also cleaved into three A*β*-like peptides [39]. As three *ε*-like cleavages are known, it is likely that APLP-1 is processed in three product lines by successive tripeptide release [30] (Figure 4). The transmembrane domain of mNotch-1 is cleaved by *γ*-secretase after ectodomain shedding to liberate NICD (S3 cleavage). NICD containing V1744 was found as the prominent species produced by S3 cleavage [55]. To date, it seems reasonable to suppose that there is a single cleavage site in S3. *γ*-Secretase also cleaves mNotch-1 at the lumen-membrane boundary (S4 cleavage) to release Notch *β* peptides (N*β*) (Figure 4) [40, 56, 57]. Fenofibrate treatment increased the proportion of N*β*25, but not that of N*β*21, which implies that N*β*25 and N*β*21 correspond to A*β*42 and A*β*40, respectively [57]. However, it is unlikely that several N*β* product lines exist in Notch processing because of the single S3 site. The production of N*β* species may not fit the tripeptide-processing model (Figure 4). CD44 is cleaved not only at the membrane-cytoplasm boundary, but also at the middle of the transmembrane domain, which results in the release of A*β*-like peptides [33, 41]. Similar to Notch, the processing of the CD44 transmembrane domain may not fit the tripeptide-processing model (Figure 4).

## 7. Conclusion and Perspectives

The tripeptide hypothesis was confirmed in the processing of the APP transmembrane domain, which accounts for the production of A*β* species. Although the physiological significance of the multiple cleavage of the transmembrane domain is unknown, it is important to illustrate the cleavage mechanisms of other *γ*-secretase substrates, because the limitation of this stepwise mechanism would help to elucidate the substrate-specific inhibition of A*β* production. As shown in Figure 4, APLP-1 may be cleaved by tripeptide release; however, Notch and CD44 do not fit this processing model [40, 41]. *γ*-Secretase is widely believed to be a promiscuous protease; however, the cleavage mechanisms of APP and Notch, at least, seem to be different (Figure 4), which indicates that *γ*-secretase distinguishes substrates during proteolysis. Perhaps absence of helix breaker glycine residues in mid-portion of transmembrane domain allows multiple S4 cleavages even after single S3 cleavage in Notch. From this point of view, uncovering the mechanisms underlying *γ*-secretase-dependent cleavage offers a basis for new therapeutic approaches that are aimed at substrate-specific A*β* inhibition.

## Abbreviations

A*β*: Amyloid *β* protein
AICD: APP intracellular domain
APP: Amyloid precursor protein
*β*CTF: Carboxyl terminal fragment of APP
DAPT: N-[N-(3,5-difluorophenacetyl)-L-alanyl]-(*S*)-phenylglycine *t*-butyl ester
FAD: Familial Alzheimer's disease
LC-MS/MS: Liquid chromatography-tandem mass spectrometry
N*β*: Notch *β* peptide
NICD: Notch intracellular domain
PS: Presenilin.

## References

[1] Haass C, Koo EH, Mellon A, Hung AY, Selkoe DJ (1992). Targeting of cell-surface *β*-amyloid precursor protein to lysosomes: alternative processing into amyloid-bearing fragments. *Nature*.

[2] Citron M, Teplow DB, Selkoe DJ (1995). Generation of amyloid *β* protein from its precursor is sequence specific. *Neuron*.

[3] Vassar R, Bennett BD, Babu-Khan S (1999). *β*-Secretase cleavage of Alzheimer’s amyloid precursor protein by the transmembrane aspartic protease BACE. *Science*.

[4] Pinnix I, Musunuru U, Tun H (2001). A novel *γ*-secretase assay based on detection of the putative C-terminal fragment-*γ* of amyloid *β* protein precursor. *Journal of Biological Chemistry*.

[5] Selkoe DJ (2001). Alzheimer’s disease: genes, proteins, and therapy. *Physiological Reviews*.

[6] De Strooper B, Saftig P, Craessaerts K (1998). Deficiency of presenilin-1 inhibits the normal cleavage of amyloid precursor protein. *Nature*.

[7] De Strooper B, König G (1999). Alzheimer's disease. A firm base for drug development. *Nature*.

[8] Wolfe MS, Xia W, Ostaszewski BL, Diehl TS, Kimberly WT, Selkoe DJ (1999). Two transmembrane aspartates in presenilin-1 required for presenilin endoproteolysis and *γ*-secretase activity. *Nature*.

[9] Struhl G, Greenwald I (1999). Presenilin is required for activity and nuclear access of notch in drosophila. *Nature*.

[10] Yu G, Nishimura M, Arawaka S (2000). Nicastrin modulates presenilin-mediated notch/glp-1 signal transduction and *β*APP processing. *Nature*.

[11] Goutte C (2002). Genetics leads the way to the accomplices of presenilins. *Developmental Cell*.

[12] Francis R, McGrath G, Zhang J (2002). aph-1 and pen-2 are required for Notch pathway signaling, *γ*-secretase cleavage of *β*APP, and presenilin protein accumulation. *Developmental Cell*.

[13] Edbauer D, Winkler E, Regula JT, Pesold B, Steiner H, Haass C (2003). Reconstitution of *γ*-secretase activity. *Nature Cell Biology*.

[14] Kimberly WT, LaVoie MJ, Ostaszewski BL, Ye W, Wolfe MS, Selkoe DJ (2003). *γ*-Secretase is a membrane protein complex comprised of presenilin, nicastrin, aph-1, and pen-2. *Proceedings of the National Academy of Sciences of the United States of America*.

[15] Takasugi N, Tomita T, Hayashi I (2003). The role of presenilin cofactors in the *γ*-secratase complex. *Nature*.

[16] Beel AJ, Sanders CR (2008). Substrate specificity of *γ*-secretase and other intramembrane proteases. *Cellular and Molecular Life Sciences*.

[17] Brown MS, Ye J, Rawson RB, Goldstein JL (2000). Regulated intramembrane proteolysis: a control mechanism conserved from bacteria to humans. *Cell*.

[18] Wolfe MS, Kopan R (2004). Intramembrane proteolysis: theme and variations. *Science*.

[19] Tolia A, Horré K, De Strooper B (2008). Transmembrane domain 9 of presenilin determines the dynamic conformation of the catalytic site of *γ*-secretase. *Journal of Biological Chemistry*.

[20] Takagi S, Tominaga A, Sato C, Tomita T, Iwatsubo T (2010). Participation of transmembrane domain 1 of presenilin 1 in the catalytic pore structure of the *γ*-secretase. *Journal of Neuroscience*.

[21] Sato C, Takagi S, Tomita T, Iwatsubo T (2008). The C-terminal PAL motif and transmembrane domain 9 of presenilin 1 are involved in the formation of the catalytic pore of the *γ*-secretase. *Journal of Neuroscience*.

[22] Takeo K, Watanabe N, Tomita T, Iwatsubo T (2012). Contribution of the *γ*-secretase subunits to the formation of catalytic pore of presenilin 1 protein. *Journal of Biological Chemistry*.

[23] Osenkowski P, Li H, Ye W (2009). Cryoelectron microscopy structure of purified *γ*-secretase at 12Å resolution. *Journal of Molecular Biology*.

[24] Citron M, Oltersdorf T, Haass C (1992). Mutation of the *β*-amyloid precursor protein in familial Alzheimer’s disease increases *β*-protein production. *Nature*.

[25] Jarrett JT, Berger EP, Lansbury PT (1993). The carboxy terminus of the *β* amyloid protein is critical for the seeding of amyloid formation: implications for the pathogenesis of Alzheimer’s disease. *Biochemistry*.

[26] Wang R, Sweeney D, Gandy SE, Sisodia SS (1996). The profile of soluble amyloid *β* protein in cultured cell media. Detection and quantification of amyloid *β* protein and variants by immunoprecipitation-mass spectrometry. *Journal of Biological Chemistry*.

[27] Citron M, Westaway D, Xia W (1997). Mutant presenilins of Alzheimer’s disease increase production of 42-residue amyloid *β*-protein in both transfected cells and transgenic mice. *Nature Medicine*.

[28] Clarke NJ, Tomlinson AJ, Ohyagi Y, Younkin S, Naylor S (1998). Detection and quantitation of cellularly derived amyloid *β* peptides by immunoprecipitation-HPLC-MS. *FEBS Letters*.

[29] Beher D, Wrigley JDJ, Owens AP, Shearman MS (2002). Generation of C-terminally truncated amyloid-*β* peptides is dependent on *γ*-secretase activity. *Journal of Neurochemistry*.

[30] Gu Y, Misonou H, Sato T, Dohmae N, Takio K, Ihara Y (2001). Distinct intramembrane cleavage of the beta-amyloid precursor protein family resembling gamma-secretase-like cleavage of Notch. *Journal of Biological Chemistry*.

[31] Sastre M, Steiner H, Fuchs K (2001). Presenilin-dependent *γ*-secretase processing of *β*-amyloid precursor protein at a site corresponding to the S3 cleavage of Notch. *EMBO Reports*.

[32] Weidemann A, Eggert S, Reinhard FBM (2002). A novel *ε*-cleavage within the transmembrane domain of the Alzheimer amyloid precursor protein demonstrates homology with notch processing. *Biochemistry*.

[33] Okamoto I, Kawano Y, Murakami D (2001). Proteolytic release of CD44 intracellular domain and its role in the CD44 signaling pathway. *Journal of Cell Biology*.

[34] Lee HJ, Jung KM, Huang YZ (2002). Presenilin-dependent *γ*-secretase-like intramembrane cleavage of ErbB4. *Journal of Biological Chemistry*.

[35] Marambaud P, Shioi J, Serban G (2002). A presenilin-1/*γ*-secretase cleavage releases the E-cadherin intracellular domain and regulates disassembly of adherens junctions. *EMBO Journal*.

[36] May P, Krishna Reddy Y, Herz J (2002). Proteolytic processing of low density lipoprotein receptor-related protein mediates regulated release of its intracellular domain. *Journal of Biological Chemistry*.

[37] Ikeuchi T, Sisodia SS (2003). The Notch ligands, Delta1 and Jagged2, are substrates for presenilin-dependent “*γ*-secretase” cleavage. *Journal of Biological Chemistry*.

[38] Takami M, Nagashima Y, Sano Y (2009). *γ*-Secretase: successive tripeptide and tetrapeptide release from the transmembrane domain of *β*-carboxyl terminal fragment. *Journal of Neuroscience*.

[39] Yanagida K, Okochi M, Tagami S (2009). The 28-amino acid form of an APLPl-derived A*β*-like peptide is a surrogate marker for A*β*42 production in the central nervous system. *EMBO Molecular Medicine*.

[40] Wanngren J, Ottervald J, Parpal S (2012). Second generation *γ*-secretase modulators exhibit different modulation of notch *β* and A*β* production. *Journal of Biological Chemistry*.

[41] Lammich S, Okochi M, Takeda M (2002). Presenilin-dependent intramembrane proteolysis of CD44 leads to the liberation of its intracellular domain and the secretion of an A*β*-like peptide. *Journal of Biological Chemistry*.

[42] Sato T, Dohmae N, Qi Y (2003). Potential link between amyloid beta-protein 42 and C-terminal fragment gamma 49-99 of beta-amyloid precursor protein. *Journal of Biological Chemistry*.

[43] Funamoto S, Morishima-Kawashima M, Tanimura Y, Hirotani N, Saido TC, Ihara Y (2004). Truncated carboxyl-terminal fragments of *β*-amyloid precursor protein are processed to amyloid *β*-proteins 40 and 42. *Biochemistry*.

[44] Qi-Takahara Y, Morishima-Kawashima M, Tanimura Y (2005). Longer forms of amyloid *β* protein: implications for the mechanism of intramembrane cleavage by *γ*-secretase. *Journal of Neuroscience*.

[45] Chévez-Gutiérrez L, Bammens L, Benilova I (2012). The mechanism of *γ*-Secretase dysfunction in familial Alzheimer disease. *EMBO Journal*.

[46] Quintero-Monzon O, Martin MM, Fernandez MA, Cappello CA, Osenkowski P, Wolfe MS (2011). Dissociation between the processivity and total activity of *γ*-secretase: implications for the mechanism of Alzheimer's disease-causing presenilin mutations. *Biochemistry*.

[47] Dovey HF, John V, Anderson JP (2001). Functional gamma-secretase inhibitors reduce beta-amyloid peptide levels in brain. *Journal of Neurochemistry*.

[48] Zhao G, Mao G, Tan J (2004). Identification of a new presenilin-dependent *ζ*-cleavage site within the transmembrane domain of amyloid precursor protein. *Journal of Biological Chemistry*.

[49] Zhao G, Cui MZ, Mao G (2005). *γ*-cleavage is dependent on *ζ*-cleavage during the proteolytic processing of amyloid precursor protein within its transmembrane domain. *Journal of Biological Chemistry*.

[50] Sato T, Tanimura Y, Hirotani N, Saido TC, Morishima-Kawashima M, Ihara Y (2005). Blocking the cleavage at midportion between *γ*- and *ε*-sites remarkably suppresses the generation of amyloid *β*-protein. *FEBS Letters*.

[51] Vetrivel KS, Cheng H, Lin W (2004). Association of *γ*-secretase with lipid rafts in post-golgi and endosome membranes. *Journal of Biological Chemistry*.

[52] Yagishita S, Morishima-Kawashima M, Ishiura S, Ihara Y (2008). A*β*46 is processed to A*β*40 and A*β*43, but not to A*β*42, in the low density membrane domains. *Journal of Biological Chemistry*.

[53] Yagishita S, Morishima-Kawashima M, Tanimura Y, Ishiura S, Ihara Y (2006). DAPT-induced intracellular accumulations of longer amyloid *β*-proteins: further implications for the mechanism of intramembrane cleavage by *γ*-secretase. *Biochemistry*.

[54] Fukumori A, Fluhrer R, Steiner H, Haass C (2010). Three-amino acid spacing of presenilin endoproteolysis suggests a general stepwise cleavage of *γ*-secretase-mediated intramembrane proteolysis. *Journal of Neuroscience*.

[55] Osawa S, Funamoto S, Nobuhara M (2008). Phosphoinositides suppress *γ*-secretase in both the detergent-soluble and -insoluble states. *Journal of Biological Chemistry*.

[56] Okochi M, Steiner H, Fukumori A (2002). Presenilins mediate a dual intramembranous *γ*-secretase cleavage of Notch-1. *EMBO Journal*.

[57] Okochi M, Fukumori A, Jiang J (2006). Secretion of the Notch-1 A*β*-like peptide during Notch signaling. *Journal of Biological Chemistry*.

